# Button battery or coin ingestion? A diagnostic challenge

**DOI:** 10.36416/1806-3756/e20260312

**Published:** 2026-09-14

**Authors:** Edson Marchiori, Bruno Hochhegger, Gláucia Zanetti

**Affiliations:** 1. Universidade Federal do Rio de Janeiro, Rio de Janeiro (RJ) Brasil.; 2. University of Florida, Gainesville (FL) USA.

An 18-month-old boy was brought to the emergency department with a dry cough and fever. His mother reported that the symptoms had begun approximately three days earlier, and that he had no other symptoms. Chest X-rays revealed a round, opaque foreign body lodged in the upper esophagus (Figure 1). Based on the foreign body’s morphological characteristics, a diagnosis of button battery (BB) ingestion was made.

**Figure 1:**
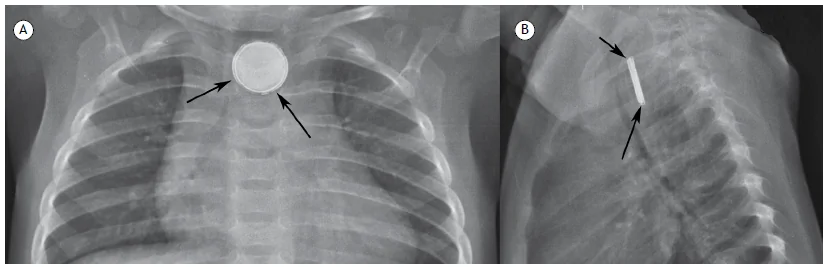
In A, an anteroposterior chest X-ray showing a circular metallic foreign body with a double peripheral rim (double-ring sign; arrows). In B, a lateral chest X-ray showing the “step-off” sign (arrows), which enabled the diagnosis of a button battery lodged in the esophagus.

BB ingestion is a pediatric emergency requiring immediate intervention. These batteries, widely used in everyday electronic devices, can cause severe and potentially fatal injuries, particularly when lodged in the esophagus. Prompt diagnosis and urgent endoscopic removal are crucial to reducing morbidity and mortality. Although most ingested BBs pass through the gastrointestinal tract and are eliminated spontaneously, impacted batteries (typically larger than 20 mm) can cause severe tissue injury.^1^ ^-^ ^4^

Diagnostic errors frequently occur when ingested batteries are mistaken for coins. Chest X-rays can help differentiate a BB from a coin. The homogeneous appearance of a coin contrasts with the “double-ring” sign (a halo of reduced radiographic density surrounding the circumference of the foreign body on the anteroposterior view) observed with a BB. This sign is caused by the negative pole (anode), which is narrower than the positive pole. On a lateral view, the “step-off” sign (a slight protrusion along one edge of the battery) may be observed, corresponding to the battery’s negative pole (anode). However, the “step-off” sign may not be visible in some newer BBs, which have a very thin profile and therefore may be radiographically indistinguishable from a coin on a lateral view.^1^ ^-^ ^4^

The primary mechanism of injury is the leakage of electrolytes from the battery. The main complications include esophageal or aortic perforation, tracheoesophageal fistula, and esophageal bleeding, among others. Complication rates tend to increase with the duration of impaction. Early endoscopic removal (ideally within 2 h of ingestion) is the treatment of choice. Surgical intervention is indicated when endoscopic removal is unsuccessful or not feasible. Even after removal, esophageal stenosis or narrowing may develop. Therefore, a post-removal follow-up plan is necessary, as some patients may require repeated esophageal dilations.^1^ ^-^ ^4^
